# Thomasville as a Winter Resort

**Published:** 1882-10

**Authors:** T. S. Hopkins

**Affiliations:** Thomasville, Ga.


### THOMASVILLE AS A WINTER RESORT.

By T. S. HOPKINS, M.D., THOMASVILLE, GA.

*Editor Atlanta Medical Register:*

DEAR SIR.—During my sojourn at Media, Pennsylvania, in the month of June last, I had the pleasure of making the acquaintance of, and being much with, the Rev. S. A. Heilner, a learned and eloquent minister of the gospel, to whom the following letter was addressed. Devoid of all sectional prejudice, like the true Christian, he feels a deep and sincere interest in everything that will redound to the public good; and knowing nothing of the claims and advantages of the pine forests of Georgia as a winter home for invalids, many of whom are to be found in his own State, I promised to make a report, addressed to him, telling what I knew as the results of personal observation, as well as what I have learned from others about it. I have done so in the letter sent you. In this letter I have deemed it not improper or unprofessional, under the circumstances, to use portions of articles of my own which have been published heretofore.

Very respectfully, and truly your friend,

T. S. HOPKINS, M.D.

*Thomasville, Ga.*, Aug. 29, 1882.

---

THOMASVILLE, GA., August 25, 1882.

*Rev. S. A. Heilner, Media, Pennsylvania:*

MY DEAR SIR.—In compliance with my promise, I write you in reference to our climate. To fully present its claims and advantages, I am necessarily compelled to be somewhat lengthy, though I trust not to such an extent as to weary your patience or trend unreasonably on your valuable time.

Our present summer to date, has been the most agreeable experienced during my long residence here. The days are as pleasant as we could wish them, the mercury seldom rising above 86° F., and the nights so delightful that there is no interference with "nature's sweet restorer," reminding us that in our morning devotions we should not forget "the man who first invented sleep."

The last winter was as remarkable for its high, as the present summer is for its low temperature; and it is of our winter climate that I wish more particularly to speak, because it more nearly concerns and interests those from a distance who seek its genial influences at that season.

Thomasville is now, without doubt, judging from the annually increasing number of invalids, becoming one of the most popular winter resorts in the whole country, and I honestly think, from personal, professional observation for a number of years, deservedly so. No subject has ever claimed or received more careful and earnest attention from the medical profession than that of winter homes for invalids. Many centuries before Christianity had an existence, the medical profession, convinced by the results of clinical experience, that the materia medica furnished but little that would not end in unsuccessful experiments and disappointed hopes, in the treatment of consumption, a disease which has vexed the minds of the profession of every age and country, and which, when medicine was in its infancy, held, as it holds to-day, the first place among the *approbria medicorum*, turned their attention to climate in search of a remedy. This, like the disease, has been the subject of perplexing inquiries, and volumes have been written upon it. The ablest men of the profession have differed widely, but doubtless honestly, as to the best climate for the consumptive. The one has sent the victim to some seaside; the other has told him to studiously avoid a residence in proximity to the coast. But these differences of opinion have, in a great measure, been removed by the results of long experience and careful observation, and the almost unanimous opinion now is, that a seaboard residence is the very worst for the consumptive. I can remember the time when Cuba and other West India islands were the resorts for consumptives from all parts of the world, but accumulated evidence against the adaptability of the climate to the disease is so overwhelming that they have ceased to be visited by that class of invalids. A host of distinguished English physicians, who having passed many years of their professional lives on these islands, and had peculiar opportunities of observing on a large scale the effects of the climate on consumptives, testify in the strongest language, not only against the adaptability, but to the actual destructiveness of the climate in such cases.

If we cross the Atlantic we find that similar objections obtain everywhere to seaboard resorts. A patient of mine here last winter, from New York, informed me that Dr. Bennett, of the Brompton Hospital for Consumptives, at London, who met her in the south of France, told her to return to America immediately, and go South; that there were no winter resorts for invalids in Europe. The same advice and opinion was given her by prominent physicians in Paris and London. These gentlemen further informed her that she had but a few months to live. She was told this five years ago. She took their advice, returned to her own country, came South, and bids fair to live many years longer. Yet the south of France used to be considered the "garden spot," the *ultima thule*, short of which there was no hope for the consumptive.

A professional experience of nearly forty years, much of which was on the seacoast of Georgia and Florida, has convinced me that our entire Southern seaboard is exceedingly objectionable as winter resorts for consumptives.

The chief objections to all seaboard resorts are, the excessive humidity, the sudden variations of temperature, and the sea winds. The prevailing winds during the winter are from the northwest and northeast. Sweeping down from northern latitudes over the sea, they become freighted with moisture and saline vapor, and reach us in a condition to irritate the bronchial membrane, aggravate the cough, and as powerful conductors of caloric, chill the body of the already enfeebled invalid, causing local congestions, resulting in hemorrhage, and speedily reducing him to a helpless condition. It is a matter of surprise that these warnings, so often uttered against seaboard resorts, are disregarded by consumptives, and that they still persist in seeking relief from climates fraught with danger, where they soon find with sorrow and regret that they have abandoned the pleasures and comforts of family and home, only to shorten their days and die among strangers.

If the invalid is to eschew the seaboard, where shall we send him? Shall we send him to the far West, with instructions to ascend and remain upon the mountains at an altitude of five or six thousand feet? We should be cautious, it seems to me, in giving such advice. We should first, by careful examination, ascertain the true condition of his lungs, and if the disease is in its incipiency, we may send him to the mountains, with a hope that the rarified condition of the air, increasing the respiratory act, would expand the lungs, develop their natural capacity and restore their impaired functions. But if the disease is advanced, with much caseous infiltration, and particularly after softening has commenced, it seems to me, with due deference to the opinions of others, that such advice would be unfortunate, if not cruel. In this stage of the disease the vital capacity of the lung is so seriously crippled that the increased frequency of respirations, upon which we might build a reasonable hope in the first case, would be futile, if not fatal in the last.

Fortunately, in this great country of ours we have sections and localities where the unfortunate invalid can find winter homes in a great measure free from all of those objections.

The beneficial influence derived from a residence in the pine forests attracted the attention of physicians in the earliest days of medicine. Hippocrates, the father of medicine, sent consumptive patients to the pine forests of Egypt, because the air was dry and healing; and Celsus, the Roman physician, in the reign of Tiberius, followed his example. Many distinguished physicians of our own country, now dead, have left to us in their valuable books, which many living fully endorse, the opinion that there is no better residence for the consumptive than in the interior of Georgia, where the immense pine forests might add the advantages of their exhalations to those afforded by the dryness and warmth of the climate; and where the dry air and uniform temperature, in connection with the aroma of the pine, would exert a highly beneficial influence. Twenty years residence in the pine forests of Southern Georgia fully warrants me in endorsing these opinions.

Having for many years, in my travels through this section of country, noticed the almost entire absence of consumption among the people, I addressed letters to a large number of physicians practicing in the district, asking them to report to me the number of cases of consumption coming to their knowledge during the previous years. I received replies from twenty engaged in active practice, and representing a population of fifty thousand, eight hundred and eighty-seven. The total number of cases reported was *three.* I have no reason to doubt the honesty of this report. A climate in which the disease so rarely occurs is certainly worthy of a trial by those who have it.

In the midst of this immense pine forest is situated the town of Thomasville, Thomas county, Georgia; two hundred miles from the Atlantic and sixty miles from the Gulf of Mexico. Thus situated, its immunity from the baneful influence of the sea winds is secured. They can reach us only after having traversed hundreds of miles of pine forest, by which they have been sifted of all saline vapor and moisture, and are consequently innocuous. The town has an altitude of three hundred and thirty feet above the sea. Its natural drainage is almost perfect, and art has supplied any deficiency. The soil is sandy, and the water pure freestone. There is no body of water within eighteen miles, and the nearest river is four miles distant.

The following is a consolidated meteorological report for the last four years, by Prof. L. S. McSwain, Volunteer Observer U. S. Signal Service at Thomasville.

Latitude, 30 50. Longitude, 84.10. Altitude, 330 feet.

| | Temperature. | Relative Humidity. |
|---|---|---|
| January | 54.39 | 65 per cent. |
| February | 55.87 | 62 " |
| March | 61.51 | 61 " |
| April | 67.35 | 60 " |
| May | 74.50 | 65 " |

| | Temperature. | Relative Humidity. |
|---|---|---|
| June | 80.02 | 63 per cent. |
| July | 81.99 | 67 " |
| August | 79.33 | 72 " |
| September | 76.12 | 71 " |
| October | 68.94 | 69 " |
| November | 58.66 | 67 " |
| December | 53.40 | 64 " |
| Annual mean | 67.78 | 65 " |

COMPARATIVE METEOROLOGY—WINTER TEMPERATURE.

| | | | |
|---|---|---|---|
| Thomasville, mean temperature | | | 54.55 |
| Aiken, S. C., | is 48.53; | colder than Thomasville. | 6.02 |
| Asheville, N. C., | is 40.60; | " " " | 13.95 |
| Jacksonville, Fla., | is 54.20; | " " " | .53 |

SPRING TEMPERATURE.

| | | | |
|---|---|---|---|
| Thomasville, temperature | | | 67.78 |
| Aiken, S. C., | is 55. ; | colder than Thomasville. | 12.78 |
| Asheville, N. C., | is 45.93; | " " " | 21.85 |
| Jacksonville. Fla., | is 67. ; | " " " | .78 |

COMPARATIVE RELATIVE HUMIDITY.

| | | | | |
|---|---|---|---|---|
| Thomasville is | | | .65 | per cent. |
| Aiken | is 64.04; | less than Thomasville. | .96 | " |
| Asheville | is 70.10; | greater than Tho'sville | 5.10 | " |
| Jacksonville | is 5.77; | " " " | 12.12 | " |

Having compared the climate of Thomasville with other noted winter resorts in the South, permit me to compare it with some of your large cities—north, east and west—during your winter and spring seasons.

WINTER TEMPERATURE.

| | | | |
|---|---|---|---|
| Thomasville | is | | 54.55 |
| Boston | is 30.37; | colder than Thomasville. | 24.18 |
| New York | is 36.50; | " " " | 18.05 |
| Chicago | is 31.63; | " " " | 22.92 |
| Cincinnati | is 39.60; | " " " | 14.97 |
| Colorado Springs | is 31.20; | " " " | 23.35 |

SPRING.

| | | | |
|---|---|---|---|
| Thomasville | is | | 67.78 |

| | | | | | |
|---|---|---|---|---|---|
| Boston | is 34.20; | colder than | Thomasville.. | | 33.58 |
| New York | is 37.10; | " | " | " | 30.68 |
| Chicago | is 35.50; | " | " | " | 32.28 |
| Colorado Springs | is 32.83; | " | " | " | 34.95 |

Examine carefully these reports, and you will be convinced that the temperature, as well as the dryness of the climate, is unsurpassed. You must not infer from them, however, that we never have any cold weather here. We do have at times heavy frosts and ice, but only for a few days, to be followed by spring-like weather. Once in the last fifteen years, we have been visited by "the beautiful snow."

The accommodations for visitors are ample. The Mitchell Hotel, M. A. Bower, proprietor, with an experienced hotel keeper from Rhode Island as manager, is one of the best equipped and best managed hotels south of the Potomac river. It can accommodate comfortably two hundred guests. It is supplied with bath rooms, billiard rooms and every necessary convenience and comfort for its guests. The kindness and attention to the sick in this house is proverbial. I speak from personal observation. In addition to the Mitchell we have the Waverly House and the Gulf House, and a number of private boarding houses, where those who are unable, or do not wish to pay the rates at a first-class hotel, can be comfortably accommodated on very reasonable terms. The roads leading to and from the town in all directions are kept in fine condition, affording delightful rides and drives among the pines.

Four livery stables furnish every description of vehicle, and are well supplied with horses for driving and riding.

We have in the town Methodist, Presbyterian, Episcopal, Baptist, and Roman Catholic churches; a male and a female college, besides a number of excellent private schools.

During the winter and spring months nearly all of the States in the Union are represented here, and Canada and Cuba aid in recruiting the "invalid brigade." When this army of invalids turn towards their far-off homes, and see their lovely vales and beautiful grass-covered hilltops overspread with winter's icy mantle, they have reason to, and do rejoice that there is one section of country on their own soil, and under the protection of their own flag, where winter quarters can be found with almost continuous sunshine, and where they will meet with a cordial welcome.

With all my faith in its preventive as well as healing powers, I would not have you believe from anything I have written that I deem the pine forests possessed of powers to resurrect. Many poor creatures come here when their hearts,

> "Like muffled drums are beating,
> Funeral marches to the grave."

To be benefitted, invalids must be able to be out of doors and take their daily sun bath. Unless they can do this they had better remain at home.

With sincere regards for you personally, and a very high appreciation of you as a minister of the gospel, I remain very truly and sincerely your friend,

T. S. HOPKINS, M.D.

